# Traction-assisted endoscopic purse-string suture for an IgG4-related inflammatory pseudotumor: a case report and literature review

**DOI:** 10.3389/fmed.2026.1897171

**Published:** 2026-10-30

**Authors:** Jinguo Liu, Chongwei Wang, Liangliang Yu

**Affiliations:** 1 Department of Endoscopy Center, Sir Run Run Shaw Hospital, Zhejiang University School of Medicine, Hangzhou, China; 2 Department of Pathology, Sir Run Run Shaw Hospital, Zhejiang University School of Medicine, Hangzhou, China

**Keywords:** endoscopic full-thickness resection, endoscopic purse-string suture, IgG4-related disease, inflammatory pseudotumor, traction assistance

## Abstract

We report a 20-year-old female with a gastric submucosal tumor at the upper body greater curvature, successfully managed by endoscopic full-thickness resection (EFTR) using a modified traction-assisted purse-string suture. The 4.0-cm wall defect was closed in 34 min (total procedure 108 min). Pathology revealed 23 IgG4 + plasma cells/HPF, an IgG4/IgG ratio of 48%, and storiform fibrosis, meeting diagnostic criteria for IgG4-related inflammatory pseudotumor (IPT). This case demonstrates that the modified technique enables secure closure of large defects in difficult locations, and emphasizes the necessity of rigorous pathological evaluation including quantitative IgG4 assessment and differential diagnosis with inflammatory myofibroblastic tumor (IMT).

## Introduction

IgG4-related disease (IgG4-RD) is a pleomorphic entity that can masquerade as a broad spectrum of malignant, infectious, and inflammatory conditions. Elevated serum IgG4 levels are a frequent, albeit inconsistent, finding. The diagnosis is often challenging due to the absence of universally validated diagnostic criteria, making histopathological evaluation the cornerstone of definitive diagnosis. The classic histological triad of IgG4-RD encompasses lymphoplasmacytic infiltration, storiform fibrosis, and obliterative phlebitis, and the disease has been documented in nearly every organ system (1–3). Gastric involvement typically manifests in two principal forms: marked gastric wall thickening or an IgG4-related pseudotumor (4). Although the latter is conventionally regarded as a gastric manifestation of IgG4-RD, it may occasionally present as an isolated gastric lesion without systemic involvement (5). Notably, three cases of gastric IgG4-related inflammatory pseudotumors (IPTs) have demonstrated normal serum IgG4 levels and an absence of other systemic IgG4-RD manifestations (6, 7). Although IPTs have been reported at numerous anatomical sites, gastrointestinal localization remains exceptionally uncommon. For isolated gastric IPTs, endoscopic resection and surgical excision constitute the primary treatment modalities.

We describe a patient with an IPT located in the greater curvature of the upper gastric body, and propose a modified traction-assisted endoscopic purse-string suture technique for defect closure following endoscopic resection. Adequate visualization of the endoscopic field is critical for both successful lesion removal and secure wound closure, particularly in cases requiring full-thickness resection. Although endoscopic resection in the gastric fundus and duodenal bulb is associated with a substantial risk of perforation (8, 9), defects at these sites are generally amenable to straightforward endoscopic exposure. By contrast, defects situated in the greater curvature of the upper gastric body pose considerable challenges in terms of both visualization and suturing, primarily owing to obscuration by overlying mucosal folds and the gravity-dependent anatomical position of this region. The modified traction-assisted endoscopic purse-string suture technique offers a distinct advantage in achieving efficient and reliable closure of such lesions in this challenging location.

## Case description

A 20-year-old female patient presented to our hospital with a one-month history of upper abdominal distension. Physical examination disclosed no abnormalities, and there was no past history of systemic inflammatory disorders. Routine laboratory testing, including serum IgG4 concentrations, was unremarkable. Gastroscopy revealed a submucosal tumor in the greater curvature of the upper gastric body (Figure 1a). Endoscopic ultrasonography (EUS) identified a hypoechoic lesion (measuring 25.5 mm × 17.7 mm) originating from the muscularis propria (Figure 1b). Subsequent contrast-enhanced abdominal computed tomography (CT) demonstrated an extraluminal mass (Figures 1c,d). The patient opted for endoscopic full-thickness resection (EFTR), as described in our previous study (10), and signed informed consent. After EFTR, exposure of the gastric wall defect proved challenging due to obscuring mucosal folds and the gravity-dependent positioning of the resection site.

**Figure 1:**
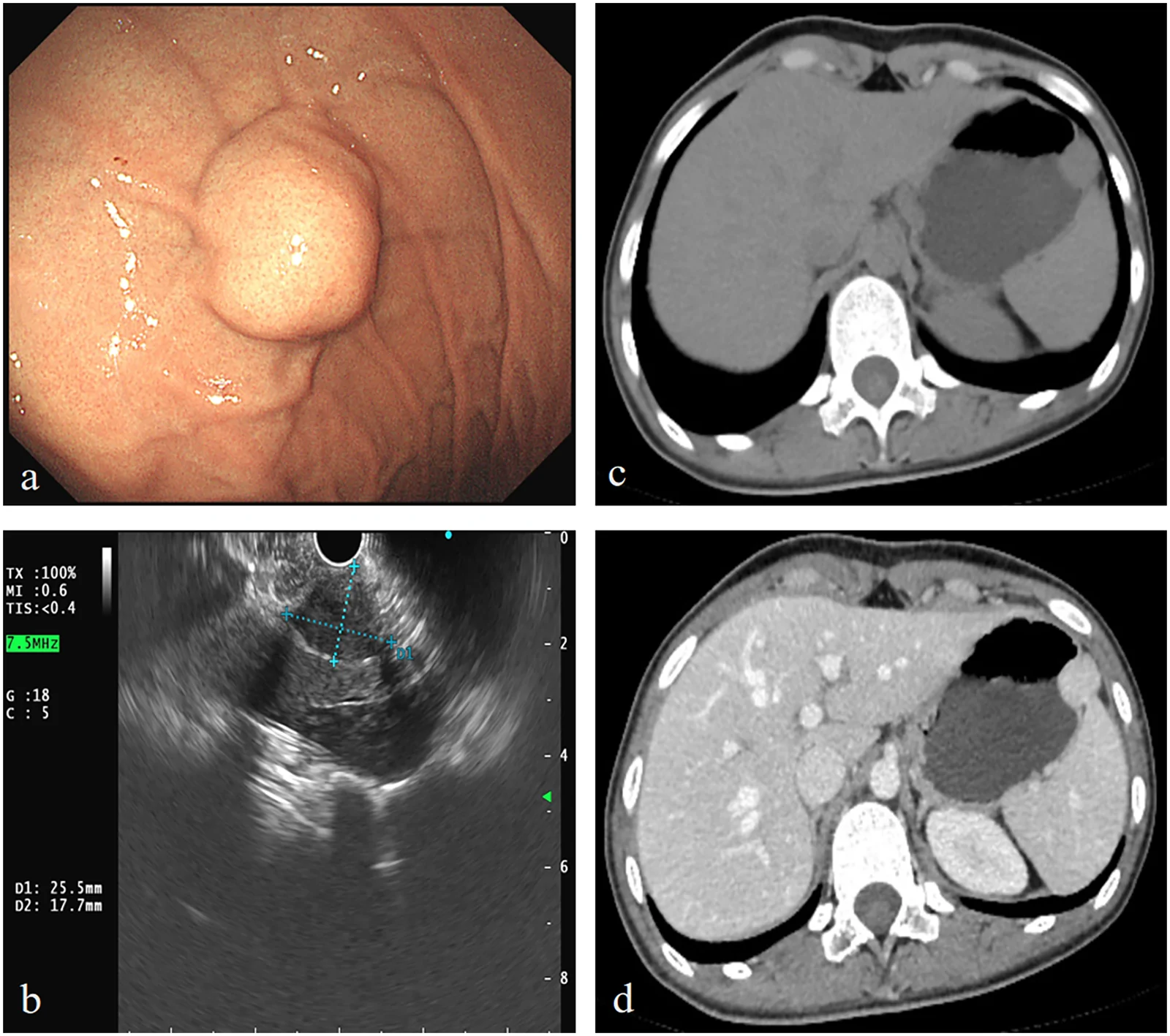
Submucosal tumor in the greater curvature of the upper gastric body. **(a)** Endoscopic view. **(b)** Endoscopic ultrasonography. **(c**, **d)** Computed tomography images.

The resulting wall defect measured approximately 4.0 cm in length. The total procedure time was approximately 108 min, with the defect closure phase lasting about 34 min. We proposed a modified traction-assisted endoscopic purse-string suture technique. Initially, a titanium clip with the dental floss was anchored to the tumor surface before full-thickness incision (Figures 2a,b). Applying traction on this dental floss pulled the tumor into the gastric lumen (Figure 2c), resulting in progressive narrowing of the defect and inward curling of the wound edges. Subsequently, the second and third titanium clips were placed to further reduce the defect size. An endoscopic purse-string suture was then performed to close the defect (Figure 2d). Crucially, the internally retracted wound configuration prevented clip inversion into the wound during suture tightening. Following complete tumor resection (Figure 2e), the closure was reinforced with additional titanium clips. Complete closure was meticulously confirmed by two methods: first, close inspection of the defect site using the transparent cap mounted on the endoscope, and second, observation of the abdominal puncture site used for pneumoperitoneum decompression, which showed no continuous gas leakage, confirming the integrity of the gastric wall closure. The patient was discharged on the fifth day without any complications.

**Figure 2:**
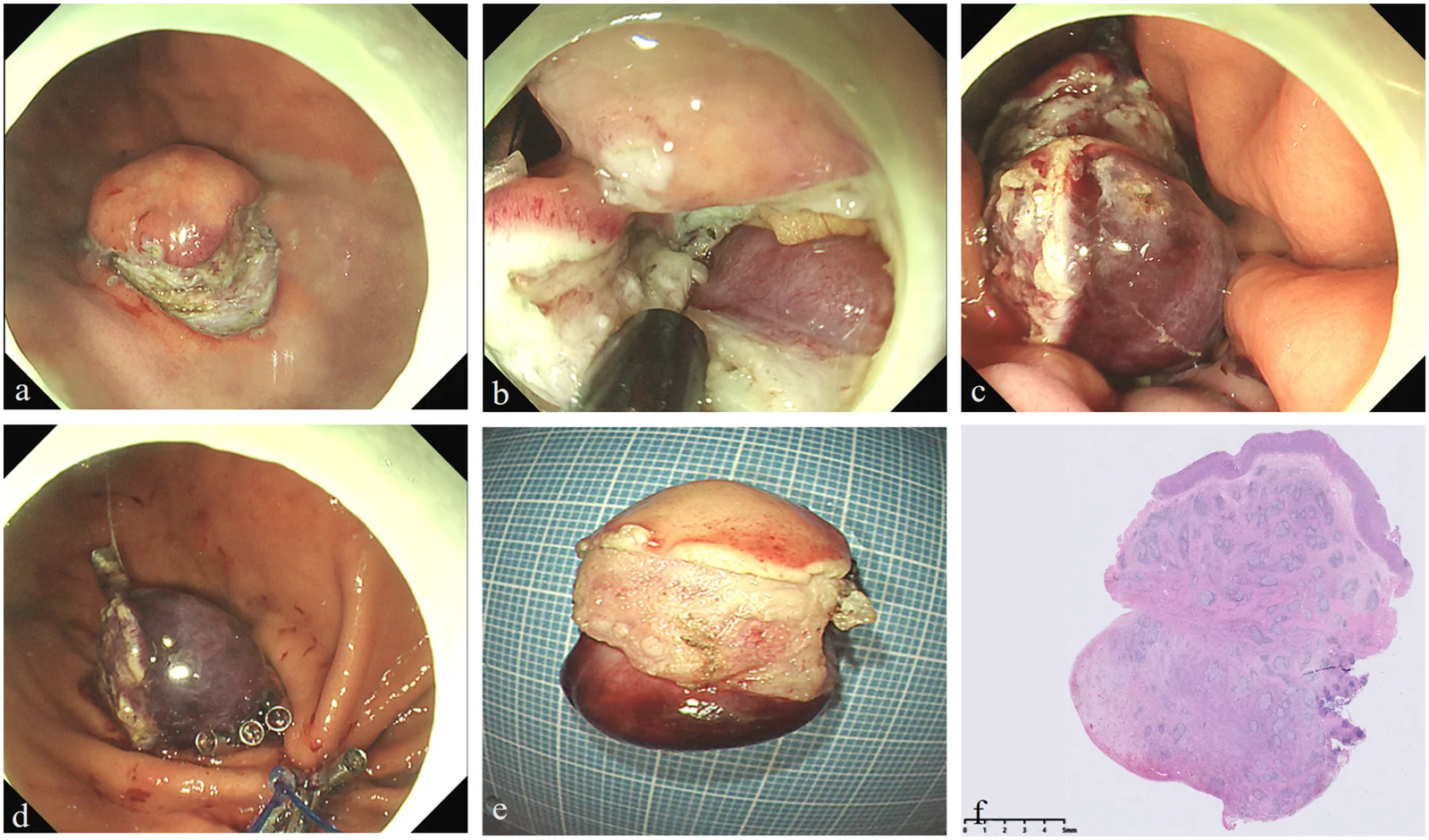
Traction-assisted endoscopic purse-string suture during endoscopic full-thickness resection. **(a)** Incision at the anal margin. **(b)** Traction and full-thickness incision. **(c)** Traction applied to retract the tumor into the gastric lumen. **(d)** Traction-assisted endoscopic purse-string suture. **(e)** Macroscopic specimen of the resected tumor (30.0 × 24.0 mm). **(f)** Hematoxylin and eosin (H&E) staining (×40).

Postoperative pathology confirmed an IgG4-related IPT (Figures 2f, 3). HE staining mainly presented with lymphoid tissue proliferation, accompanied by plasma cell infiltration and storiform fibrosis. Immunohistochemistry revealed approximately 23 IgG4-positive plasma cells per high power field (HPF) and an IgG4/IgG ratio of 48%. Although storiform fibrosis was observed, obliterative phlebitis was notably absent in the examined sections. These findings, while meeting the diagnostic threshold for IgG4-RD, highlight the heterogeneity of histological manifestations in gastric lesions. It is crucial to distinguish IgG4-related IPT from inflammatory myofibroblastic tumor (IMT), as the latter is a true neoplasm with malignant potential, often driven by ALK gene rearrangements, whereas the former is a non-neoplastic fibro-inflammatory lesion. Postoperative pathology predominantly showed lymphoid tissue proliferation accompanied by plasma cell infiltration and storiform fibrosis, which differed from the characteristic spindle cell proliferation seen in IMT. In addition, the negative ALK immunostaining, a markedly increased IgG4 + cell count (23/HPF), and an elevated IgG4/IgG ratio (48%) strongly favored a diagnosis of IPT over IMT.

**Figure 3:**
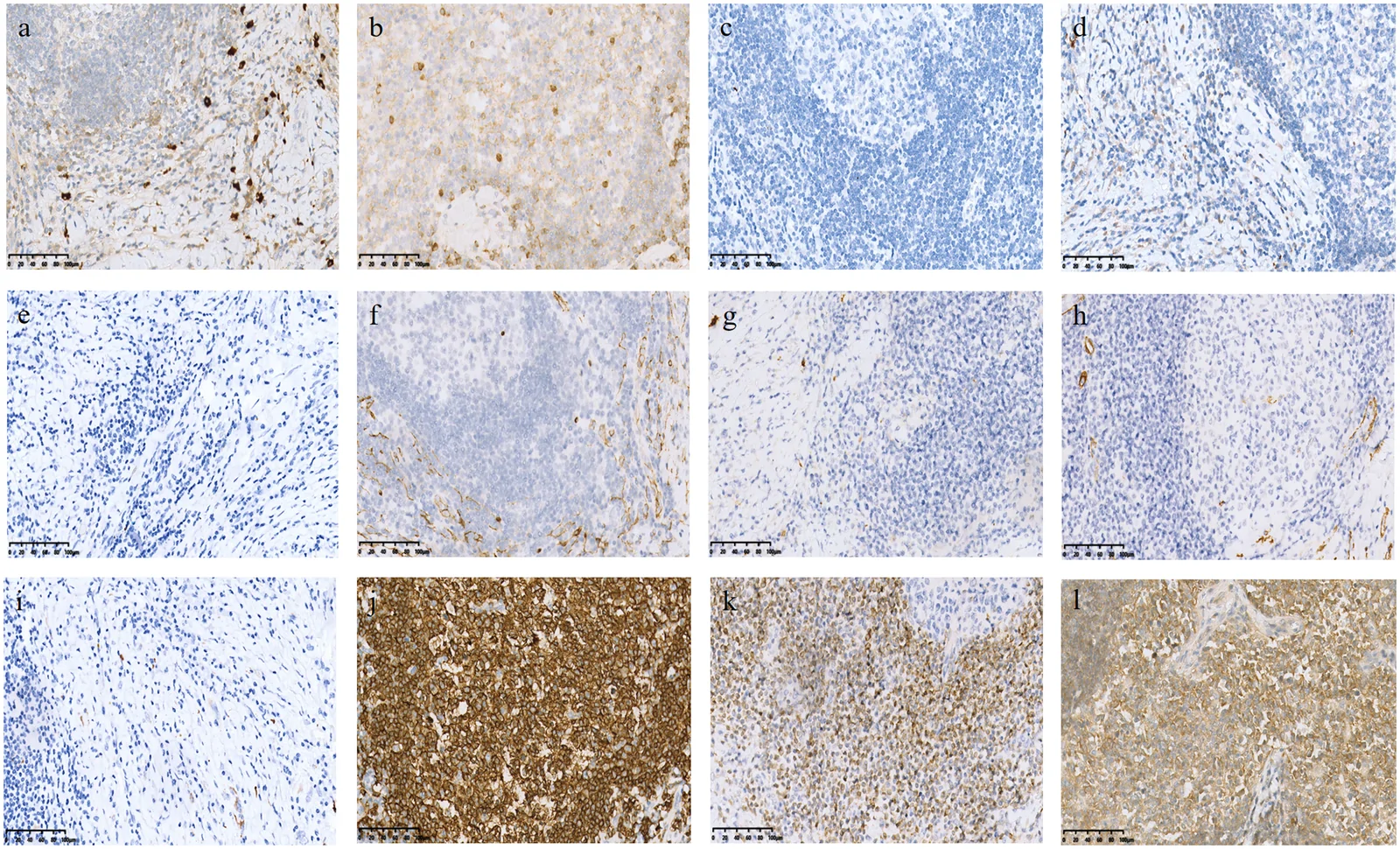
Immunohistochemical analyses. **(a**, **b)** Immunohistochemistry of IgG4 **(a)** and IgG **(b)** showing a markedly increased IgG4 + cell count (23/HPF) and an elevated IgG4/IgG ratio (48%). **(c)** Negative ALK immunostaining. (**d-f**) Immunohistochemistry of CD117 (-, d), DOG-1 (-, e), and CD34 (endothelium +, f). (**g**) Negative S100 immunostaining. (**h-i**) Immunohistochemistry of SMA (focal spindle cells +, h) and Desmin (-, i). (**j-l**) CD20 **(j)**, BCL-2 **(k)**, and BCL-10 **(l)** showing positive expression in lymphoid tissue. × 200 magnification.

Follow-up gastroscopy at 6 months revealed mucosal healing with residual nylon endoloop and several retained titanium clips (Figure 4). Postoperative monitoring still showed normal serum IgG4 levels, and abdominal CT revealed no evidence of disease recurrence or extragastric manifestations.

**Figure 4:**
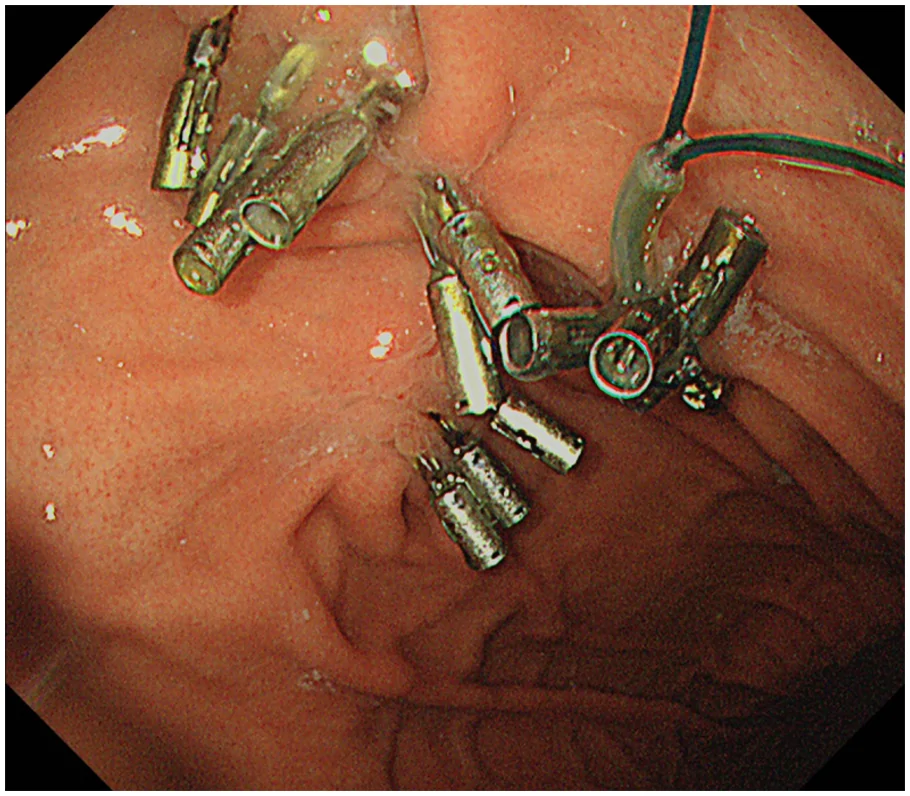
Follow-up gastroscopy showing mucosal healing with residual nylon endoloop and titanium clips.

### Instrument specifications

Titanium clips: rotatable repetitive opening and closing soft tissue clips, opening width >10 mm.

Nylon endoloop: Loop-20 mm.

Electrosurgical knife type: disposable high-frequency water-injectable incision knife, 2 mm tip.

Generator setting: ERBE VIO 200S.

## Discussion

Gastric IgG4-related IPT is generally considered a gastric manifestation of IgG4-related disease, often presenting as a solitary lesion (5) and occurring in the absence of other organ involvement (6, 7). IPT is a non-neoplastic proliferative lesion resulting from chronic inflammatory responses. Gastrointestinal involvement is rare, with gastric tumors in young girls being the most frequently described presentation (11, 12). The pathogenesis of IPT remains unclear but may be related to immune-mediated inflammation secondary to infection. Although approximately 50% of orbital pseudotumor cases achieve complete resolution with steroid therapy (11), gastric IPTs usually require pathological diagnosis following surgical or endoscopic resection. Gastric IPTs often display features suggestive of malignancy, including ulceration, transmural wall infiltration, and extragastric extension (11). Compared with IPT, IMT has malignant potential. For instance, Zou et al. reported a case of a 29-year-old woman with a gastric IMT that exhibited local invasion into the esophagus and spleen (13). Gastric IPTs should be considered in the differential diagnosis of gastric tumors with invasive features, especially in young patients. Definitive diagnosis relies on pathological evaluation, and complete local resection appears sufficient in most cases given the low malignant potential.

Including the present case, a total of 13 gastric IgG4-related inflammatory pseudotumors (6, 7, 14–21) have been reported (Table 1), with an age range of 20–75 years, no significant sex predilection, and a predilection for the gastric body. Almost all cases showed abundant IgG4-positive plasma cell infiltration with an IgG4/IgG ratio >40%, meeting the diagnostic criteria for IgG4-RD; however, serum IgG4 levels were normal in most patients, underscoring the essential role of histological examination. Approximately half of the cases presented as isolated gastric involvement, suggesting that the disease can occur as a solitary gastric manifestation. While surgical resection was the predominant treatment, endoscopic resection is a feasible alternative.

**Table 1:** Summary of previously reported gastric IgG4-related IPTs.

Author [Year]	Age (Sex)	Location	Serum IgG4	Tissue IgG4+/HPF	IgG4/IgG	Other IgG4-RD	Treatment
Novatti et al. [2022] (14)	67(M)	Pylorus	↑	>10	>40%	Yes	Prednisone Azathioprine
Piyade et al. [2020] (15)	44(M)	Body	-	42	-	-	Surgery
Cheong et al. [2016] (16)	27(F)	Fundus	Normal	102	25.3%	No	Surgery
Woo et al. [2016] (17)	48(F)	Body	-	210	85%	No	Surgery
Kim et al. [2012] (6)	59(F) 54(F)	Body	Normal	>50	-	-	Surgery
Chetty et al. [2011] (18)	45(F)	Fundus	Normal	121	78%	-	Surgery
	60(M)	Antrum	-	99	84%	-	Surgery
Ortuño et al. [2017] (19)	67(M)	Body	Normal	>50	>40%	-	Surgery
Rollins et al. [2011] (20)	75(F)	Body	Normal	39	-	-	Surgery
Na et al. [2012] (7)	56(M)	Body	-	102	80–90%	-	Endoscopic resection
Bateman et al. [2012] (21)	73(F)	Body	-	>100	80–90%	-	Surgery
Present study	20(F)	Body	Normal	23	48%	No	Endoscopic resection

Limitations regarding IMT exclusion. Although ALK immunohistochemistry was negative in the lesional cells, we acknowledged that ALK negativity by immunohistochemistry alone could not entirely exclude IMT. Ideally, ALK FISH or RNA-based fusion analysis would provide additional molecular confirmation. However, ALK FISH typically required freshly collected tissue or specific transport conditions that were not available at the time of specimen procurement. RNA-based fusion testing was not performed because a validated RNA fusion detection platform was not available at our institution at the time of diagnosis. The diagnosis therefore rested on the integrated morphological and immunohistochemical findings, including a lymphoplasmacytic-predominant infiltrate with storiform fibrosis, absence of the compact spindle cell fascicles characteristic of IMT, a markedly elevated IgG4 + plasma cell count (23/HPF), and an IgG4/IgG ratio of 48%. These findings strongly favored IgG4-related IPT over ALK-negative IMT. Future studies with molecular profiling capabilities are warranted to further refine the differential diagnosis between these entities.

Traction assistance provides a practical approach for EFTR. Double-traction assistance has been employed in EFTR for the resection of a gastric submucosal tumor (22). A modified endoscopic purse-string suture technique using dental floss traction has been applied to manage a duodenal defect (9). Furthermore, a novel endoscopic closure method termed “internal traction-assisted suspended closure” has been used to close post-EFTR defects (23). These traction-assisted techniques reduced the difficulty of defect closure and shortened the procedural time. As described in our previous study (10), experienced endoscopists completed the procedure after EFTR in 25–83 min by a single traction combined with pre-clamping technique. The current approach introduces a critical modification: In our previous method, traction and pre-clamping were used solely to facilitate lesion closure. Here, we strategically combine this strategy with a subsequent endoscopic purse-string suture. This synergy of traction, pre-clamping, and purse-string suture allows for the secure closure of large defects in anatomically challenging locations-a limitation of previously reported purse-string or single-traction techniques. Prior studies have reported operative durations of 28–89 min using clip-assisted traction (24) and 25–130 min with snare-assisted methods (25), with no severe adverse events in either series. Notably, snare traction was associated with significantly shorter operative times and a lower incidence of intraoperative bleeding compared with conventional endoscopic EFTR (53.6 ± 16.6 min vs. 67.7 ± 33.4 min) (26). Complete inverted closure has been observed for both seromuscular purse-string sutures and over-the-scope clips (OTSCs) following EFTR, with OTSCs demonstrating greater closure strength than purse-string sutures (27). However, a single OTSC is suitable only for defects of limited size. Our modified traction-assisted endoscopic purse-string suture technique overcomes this size limitation by combining traction with pre-clamping to narrow the defect. While traction pulls the tumor into the gastric lumen to improve tumor exposure and enable resection, it simultaneously induces progressive defect narrowing and inward curling of the wound edges. This internally retracted configuration subsequently prevents clip inversion into the defect during suture tightening.

## Data Availability

The original contributions presented in this study are included in this article, further inquiries can be directed to the corresponding author.
